# Conception and Theoretical Study of a New Copolymer Based on MEH-PPV and P3HT: Enhancement of the Optoelectronic Properties for Organic Photovoltaic Cells

**DOI:** 10.3390/polym14030513

**Published:** 2022-01-27

**Authors:** Mariem Ltayef, Maha M. Almoneef, Walid Taouali, Mohamed Mbarek, Kamel Alimi

**Affiliations:** 1 Research Laboratory, Asymmetric Synthesis and Molecular Engineering of Materials for Organic Electronic (LR18ES19), Monastir University, Monastir 5000, Tunisia; ltayefmeriem@gmail.com (M.L.); tawali_walid@yahoo.fr (W.T.); mohamedmbarek99@yahoo.fr (M.M.); kamel.alimi@fsm.rnu.tn (K.A.); 2 Department of Physics, College of Science, Princess Nourah Bint Abdulrahman University, Riyadh 11671, Saudi Arabia

**Keywords:** charge transfer, DFT, optical transient, optoelectronic, solar cells

## Abstract

A new copolymer has been studied, which is formed by Poly(2-methoxy-5-(2-ethyl-hexyloxy)-1,4-phenylene-vinylene) (MEH-PPV) and poly(3-hexylthiophene) (P3HT). The choice of these π-conjugated polymers was based on their semiconductor characters and their great applicability in electronic organic devices. The structure and vibrational and optoelectronic properties were simulated by calculations based on DFT, TD-DFT, and ZINDO. This material shows original and unique properties compared to the basic homopolymers. Thus, the obtained results reveal that this copolymer can be mixed with the (6,6)-phenyl C61 butyric acid methyl ester (PCBM) to give existence to a new composite that can be used as an active layer for an organic solar cell.

## 1. Introduction

The optoelectronic devices have been tremendously studied in order to upgrade and develop their properties. For the same reason, it has been attempted to find materials that could be used as active layers in these optoelectronic devices. The new generation of the components is highly valued because they are based on organic materials that offer not only flexibility and lightweight but also large surface and potentially low-cost devices [1]. Conjugated polymers are considered as one of the most successful and promising materials to be used as active layers in organic optoelectronic devices, such as in organic light-emitting diodes (OLEDs) and organic solar cells (OSCs) [2,3,4]. 

A deeper understanding of the structure-properties correlation is required as the main purpose of our study. In order to attain the objective, theoretical calculations based on DFT are employed to design and to model a new copolymer derived by Poly(2-methoxy-5-(2-ethyl-hexyloxy)-1,4-phenylene-vinylene)(MEH-PPV) combined with the poly(3-hexylthiophene) (P3HT). The choice of these polymers is based on their close optoelectronic properties and their conductor characters, where their electrical conductivities are equal to 8.8 × 10^−8^ S/m and 3.1 × 10^−5^ S/cm, respectively, for MEH-PPV [5] and P3HT [6]. Also, these two polymers are the most tested in organic electronics. Hence, the testing combination of the two polymers leads to a new copolymer with optimal properties of the two basic homopolymers. The interesting photoconductive properties, such as the capacity of absorbance in the visible, the low gap, and the high charge transport, are features that make these polymers mostly used in the optoelectronic fields [7,8,9]. 

Similar simulations with DFT were performed to evaluate the different properties of some conjugated polymers and also to determine the effects of the charge transfer in the donor-acceptor systems for an organic solar cell. As examples, we can note the study developed by Peng Song’s team, which is based on the investigation of the photoinduced charge transfer in the (P3HT: PCBM) systems and their effects on the photophysical properties [10]. For the same system, Liu’s group [11] and Debkumar’s team [12] made a detailed theoretical study that describes the structure and the optoelectronic parameters of the different oligomers, which are indicated that the variation of the energy depends on the number of monomeric in each oligomer. Moreover, Mamduh J. Aljaafreh et al. investigated the theoretical optoelectronic properties of an optimized structure of a copolymer that is based on MEH-PPVB and compared the obtained results with the experimental [13].

The new copolymer MEH-PPV-P3HT has not been synthesized, so our strategy is to study the properties derived from the combination of MEH-PPV and P3HT and to deeply describe their optoelectronic properties. In this way, this study can be helpful to better understand the relationship between the structure and the properties of the results copolymer and their application as an active layer of solar cells. 

For more insights into the effects of the coupling on the conformational, optoelectronic, and vibrational parameters, the modeling of these polymers should be carried out.

## 2. Computational Details 

The structures of the basic homopolymers and their derived copolymers were modeled using the density functional theory (DFT) [14,15]. The geometric optimization was carried out using the functional hybrid B3LYP method [16,17,18] and the base set 6-31G (d, p) [19]. The vibrational and the electronic properties, such as HOMO (Highest Occupied Molecular Orbital) and LUMO (Lowest Unoccupied Molecular Orbital) energetic levels, were calculated by the same method. To determine the coupling sites between the monomers, an oxidized optimization was performed to evaluate the spin density values in each monomer. These latter represent the electronic density distribution in the cation radical from the one-electron (+1) charged [20,21,22]. Afterward, the optical absorption spectra were simulated by two different methods, which are time dependant-DFT [19] using the same base set, but the functional hybrid was CAM-B3LYP [23,24,25] and ZINDO. These methods are also used to model the photoluminescence spectra, which were obtained by a re–optimization using the CIS method [26] with an STO-3G base set. The simulation of these optical spectra considers the presence of chloroform solvent. Furthermore, for the excited state, the electronic transition assignments and oscillator strengths were calculated utilizing the SWIZARD program [27]. All these theoretical calculations were accomplished with the Gaussian 09 program [28]. 

## 3. Results and Discussion 

### 3.1. Conformation Studies of MEH-PPV and P3HT

**Figure 1:**
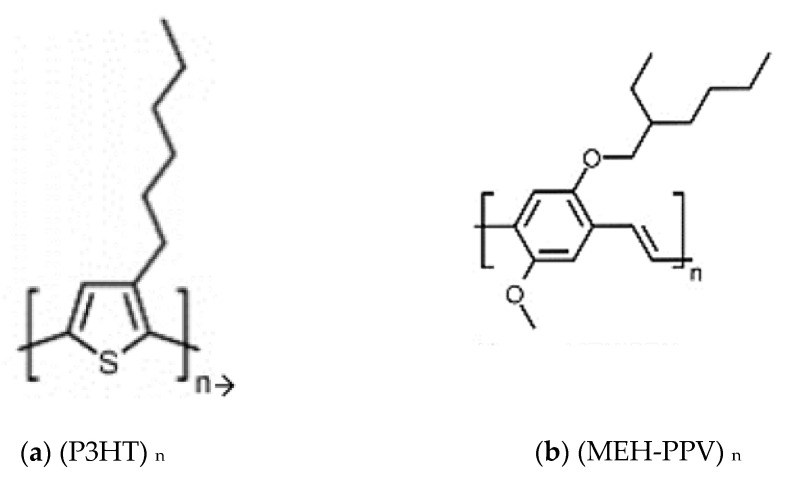
Basic structures of (**a**) P3HT and (**b**) MEH-PPV polymers.

Figure 1 shows the basic structures of polymers P3HT and MEH-PPV. 

The first step to model these polymers is the optimization in the ground state of their structures by varying the number of monomeric units *n* (*n* = 1, 2, …) in order to find the most stable geometries. The choice of conformer can be verified equally by the maximum absorption given by TD-DFT/CAM-B3LYP/6-31G (d, p) methods and ZINDO, comparing them to the experimental.

The optoelectronic properties obtained by these calculations are summarized in Table 1 and Table 2.

From the results shown in these tables, it is clear that the increase of the number of monomers leads to the decrease of the HOMO level energy and the elevation of the LUMO level energy. Consequently, the energy of the electronic gap $E_{g}^{é\mathrm{le}}$ is diminished where $E_{g}^{é\mathrm{le}}$= E_HOMO_ − E_LUMO_. 

Subsequently, from the two tables, we notice that the electronic gap energy of tetramer 4MEH-PPV (2.7 eV) and of the homopolymer 6P3HT (2.7 eV) are close to the experimental values of the gap energy of the polymers MEH-PPV and P3HT, which are equal to 2.3 eV [29] and 2 eV [30], respectively.

Furthermore, the experimental values of the maximum absorption for tetramer MEH-PPV and P3HT in chloroform solution are around 431 nm [31] and 451 nm [32], respectively. The simulation carried out by the TD-DFT indicates that the maximum of the absorption is 425 nm and 470 nm, respectively, for the 4MEH-PPV and 6P3HT. These values are closer to the experimental values than the ones given by the ZINDO, which suggests that the TD-DFT method is more reliable than the ZINDO method.

**Figure 2:**
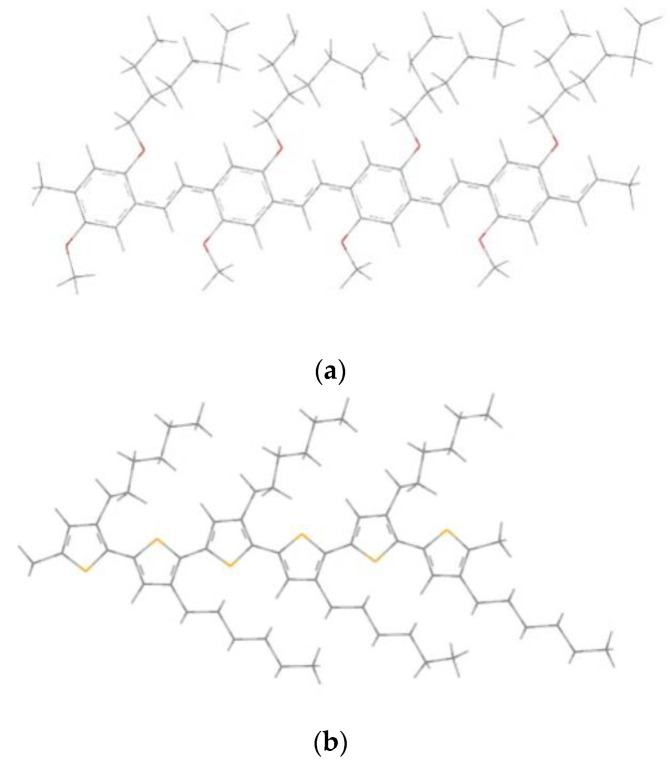
Geometrics structures of (**a**) 4MEH-PPV and (**b**) 6P3HT.

Relying on these results, we can choose the 4MEH-PPV as a conformer of the tetramer MEH-PPV and the homopolymer 6P3HT as a conformer of the polymer P3HT. Hence, the chain lengths of our polymers are formed by four monomeric units for the MEH-PPV and six monomeric units for the P3HT, and the geometric structures of these polymers are shown in Figure 2.

### 3.2. Conformation Studies of Copolymers 4MEH-PPV-6P3HT

During the conformation of these homopolymers, a study of the different architectures of their derived copolymers was carried out by the spin density and dihedral angle (scan). Knowing that the scan is the variation of the relative energy via the potential energy surface (PES) in the function of the angle of torsion, which is varied between 0 and 180° with a step of 20°, we define the most stable conformer of each polymer by checking their minimum relative energy. 

**Figure 3:**
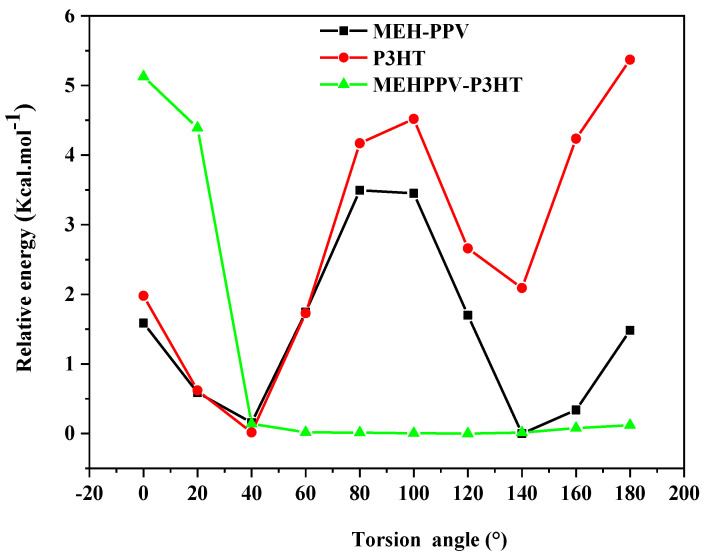
Energy curve of MEH-PPV, P3HT, and MEHPPV-P3HT.

Figure 3 illustrates the obtained results and shows that the PES of MEH-PPV presents two minimum relative energies, which correspond to the dihedral angles 40° and 140°; however, the P3HT has only one minimum energy corresponding to the angle 40°. Furthermore, the dihedral angle between MEH-PPV and P3HT is equal to 60°, which is related to the torsion angle of the block copolymer. This twisted structure reveals that the torsion angle between the two basic polymers can affect the structural and electronic properties [33].

**Figure 4:**
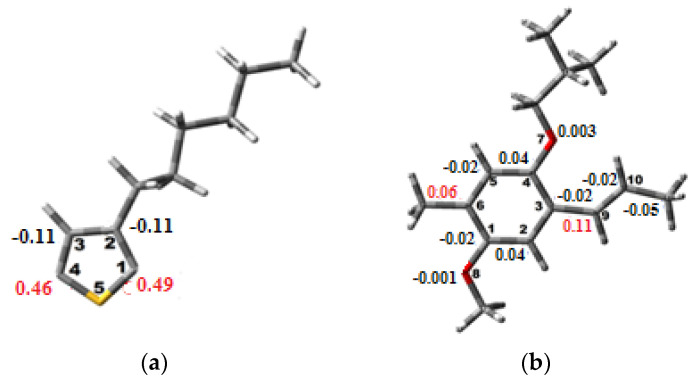
Spin density of (**a**) P3HT and (**b**) MEH-PPV.

To assess the spin density of each polymer, an optimization in the oxidized state was carried out [34]. Notably, the highest values of the density designate the coupling sites [35,36,37]. In fact, the results of these calculations shown in Figure 4 reveal that, for the P3HT, the high spin densities are located in the first and fourth carbon positions, whereas those for the MEH-PPV are located in the sixth and ninth carbon positions.

**Figure 5:**
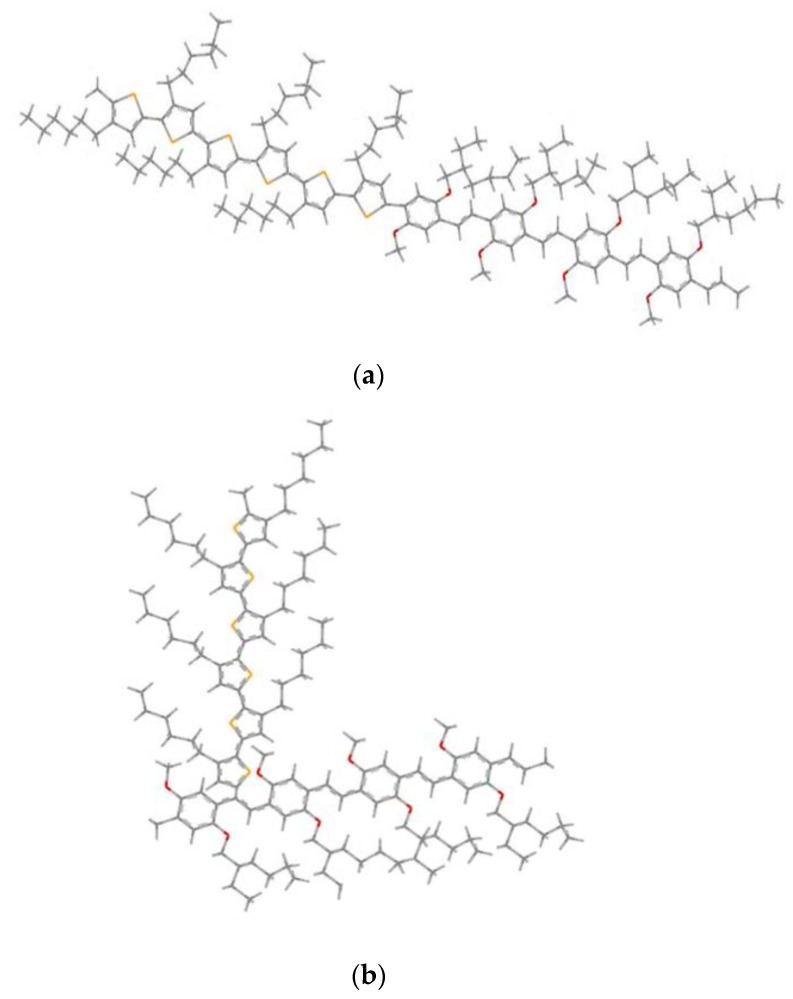
Geometrics structures of 4MEH-PPV-6P3HT: (**a**) Block copolymer and (**b**) graft copolymer (Ramif).

We propose two model structures; the first one is formed by the linear assembly of a block 4MEH-PPV with a block 6P3HT (Figure 5a). The second model structure, illustrated in (Figure 5b), is shaped by the graft of segments 6P3HT in the ninth carbon position of MEH-PPV.

### 3.3. Vibrational Properties

**Figure 6:**
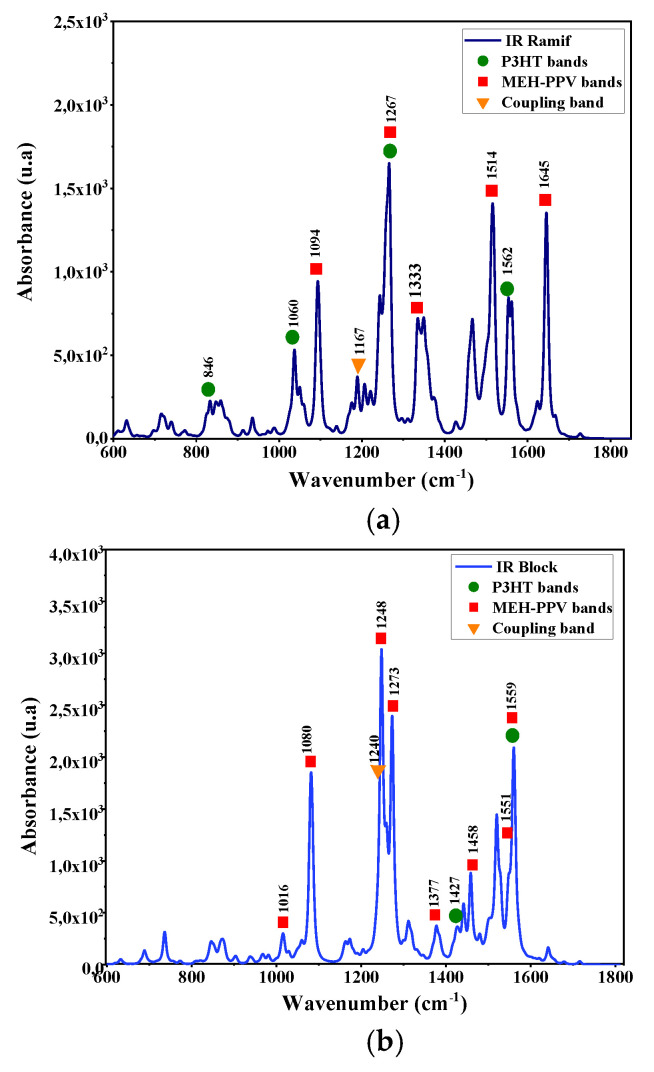
Infrared spectra simulated by DFT/B3lYP/6–31G (d, p) for: (**a**) the graft copolymer (Ramif) and (**b**) block copolymer.

**Figure 7:**
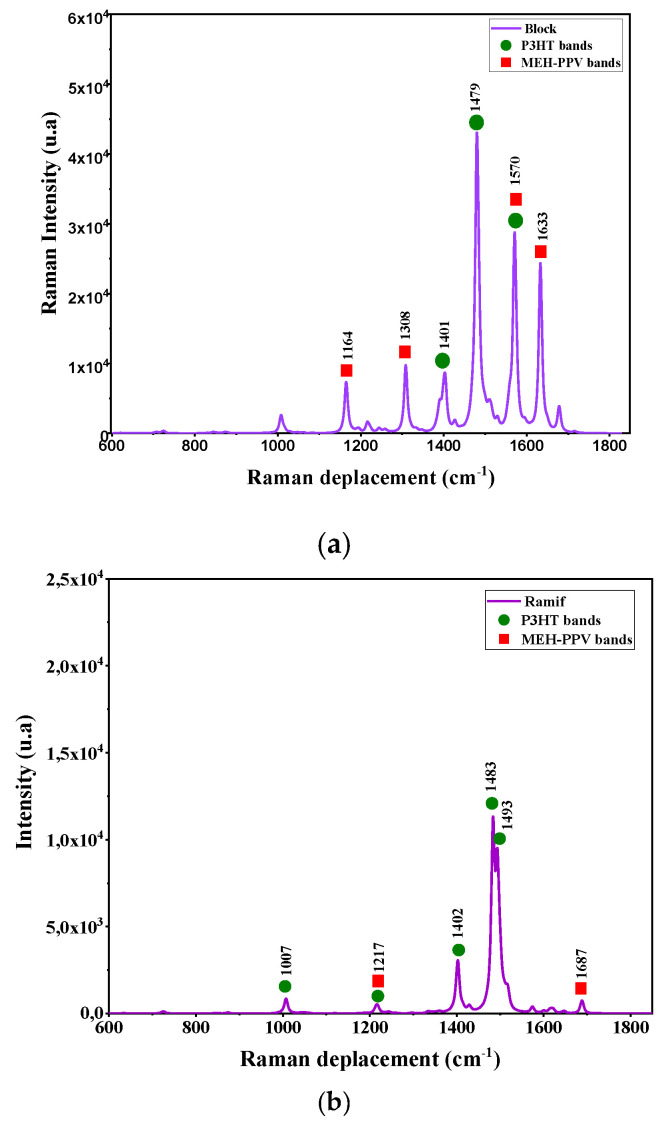
Raman spectrum simulated by DFT/B3lYP/6–31G (d, p) for: (**a**) the graft copolymer (Ramif) and (**b**) block copolymer.

The vibration modes of these copolymers are deduced by a simultaneous simulation of Infrared and Raman spectra. These spectra are presented in Figure 6 and Figure 7. The corresponding IR and Raman vibrational modes are listed in the Table 3 and Table 4.

The analysis of these spectra shows the presence of characteristic bands of the vibration modes of the two basic polymers MEH-PPV and P3HT. Moreover, the appearance of coupling bands of MEH-PPV-P3HT corresponds to the frequencies 1167 cm^−1^ (C–C symmetrical stretching between vinyl and thiophene group) and 1265 cm^−1^ (asymmetrical stretching of phenyl and thiophene group) respectively in the graft (Ramif) and block copolymer.

To compare the simulated Raman spectra of these copolymers with those of P3HT and MEH-PPV oligomers, we referred to theoretical studies of these two polymers using the DFT method. It is noted that the Raman spectrum of P3HT is close to that of Ramif copolymer, and the characteristic bands of P3HT presented in this spectrum are also almost in agreement with that of P3HT oligomers [38,39,40]. This latter can confirm the great contribution of the P3HT blocks. Moreover, the characteristic peaks of MEH-PPV that appear in the spectrum of copolymers are approximate to those donated by the Raman spectra of P3HT oligomers, which correspond to 1632 cm^−1^, 1692 cm^−1^ (C=C stretching modes in vinylene group) [41,42]. 

### 3.4. Electronic Properties 

Table 5 sums up the electronic properties of the copolymers MEH-PPV-P3HT.

The obtained results explain the coupling effects of the two basic homopolymers 4MEH-PPV and 6P3HT. To compare the graft copolymer (Ramif) with 4MEH-PPV, the graft of the P3HT on the chain of MEH-PPV reveals the destabilization with the increasing of the HOMO level of the energy from 4.29 eV to 4.54 eV and the LUMO level from 1.52 eV to 1.82 eV. These variations imply the decreasing of the band gap to be equal 2.72 eV, and this reduction subsequently explains that there is a charge transfer during this copolymerization. Nevertheless, the electronic properties of the copolymer Ramif are very proximate to that of P3HT; this can be explained by the rupture of MEH-PPV conjugation at the vinyl group, which implies the major contribution of P3HT.

Additionally, for the block copolymer, the variation of HOMO and LUMO level energies entail a diminution of the gap energy by 0.3 eV and 0.1 eV compared with that of 4MEH-PPV and 6P3HT, respectively. These results interpret the increase of the length of the chain, which implies the higher charge transfer.

**Figure 8:**
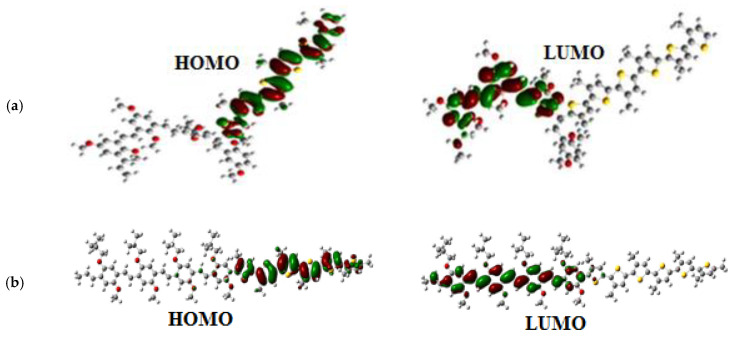
Molecular orbitals of HOMO and LUMO levels of: (**a**) the Ramif copolymer and (**b**) the Block copolymer.

The charge distribution in these copolymers was determined by the analysis of the molecular orbitals of HOMO and LUMO levels, which are shown in Figure 8. The electron density in the HOMO level is localized in the P3HT units; however, the electron density in the LUMO level is situated in the MEH-PPV units.

### 3.5. Optical Properties 

#### 3.5.1. Absorption Spectrum

**Figure 9:**
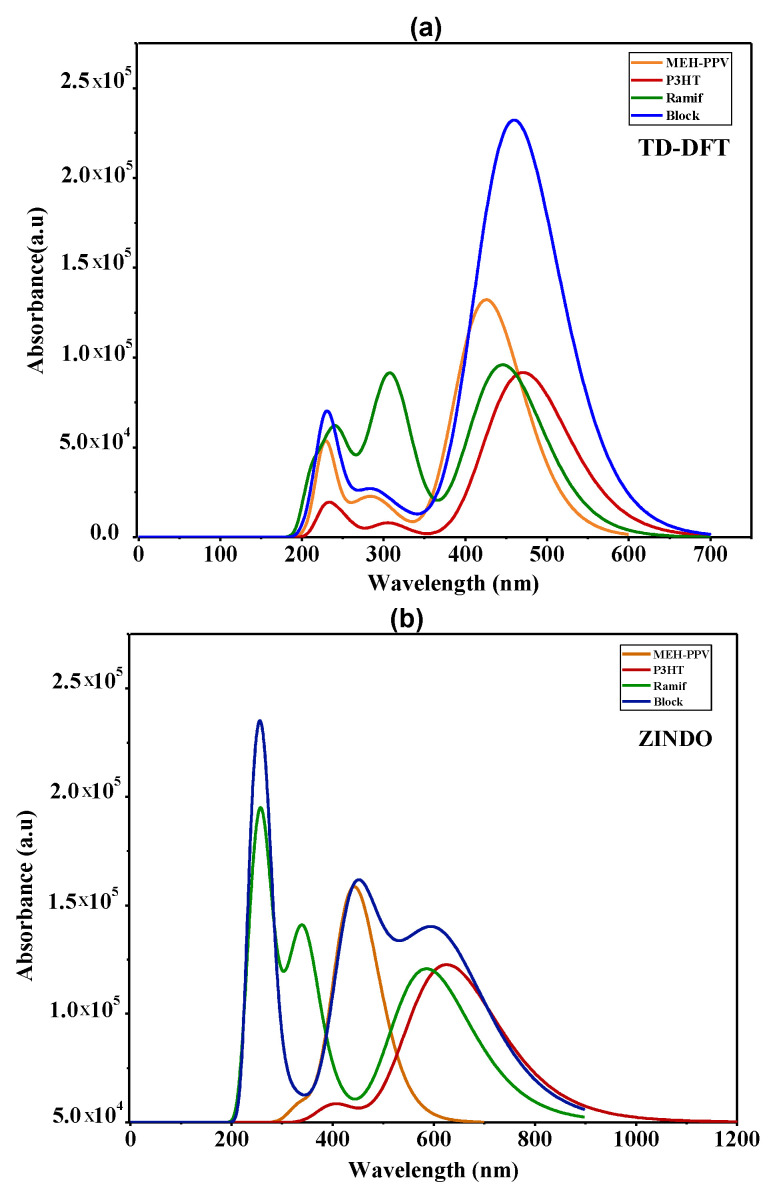
The absorption spectra of MEH-PPV, P3HT, and Block and Ramif copolymer; (**a**) TD-DFT calculations, (**b**) ZINDO calculations.

The absorption spectrum of the copolymers and the basic homopolymers simulated by TD-DFT and ZINDO in chloroform solution are presented in Figure 9. Table 6 summarizes the characteristics and the electronic assignments of the maximum absorption bands of these polymers. 

After comparing the absorption spectra of these copolymers and those of the two basic homopolymers given by the two calculation methods, we can deduce the coupling effect on the optical properties.

For the absorption spectra of the graft copolymer (Ramif), we notice that the two absorption bands, which are attributed to the *n*→π* transitions (located between 200 nm and 350 nm), are the combination of two contributions (MEH-PPV + P3HT). 

However, the band that is assigned to the π→π* transition, which corresponds to the maximum of absorption, exhibits a blueshift from that of P3HT and a redshift from that of MEH-PPV, where this shift refers to a low-charge transfer between the two polymers. It is clear that there is no enlargement of the spectrum of this copolymer, which verifies our hypothesis of rupture of MEH-PPV conjugation as well as the contribution and the dominance of P3HT in the π→π* transition.

Subsequently, for the block copolymer, the absorption spectra given by the two methods show that the band, which is assigned to the π→π* transition, undergoes an enlargement, which spreads and covers the whole absorption range of two basic homopolymers. Subsequently, it is noted that there is an increase in absorbance that reflects the hyperchromic effect compared to those of MEH-PPV and P3HT. This increase generates a decrease in the energy of the optical gap, the estimate of the energy of which is determined using the following formula [43]:

(1) $$\;\;{\;E}_{g}=\frac{1240\;}{\lambda }$$

where λ is the intersection between the linear part of spectra and the abscissa axis.

Focusing on Table 7, we notice that the optical gap of this copolymer is lower than that of MEH-PPV and P3HT. Indeed, this decrease explains the higher charge transfer along the chain; subsequently, this assembly leads to the increase in the conjugation length that is due to the combination of the two blocks contributions of the MEH-PPV and P3HT, which clearly explains the enlargement of the absorption spectrum compared to the two basic homopolymers

#### 3.5.2. Photoluminescence Spectrum

**Figure 10:**
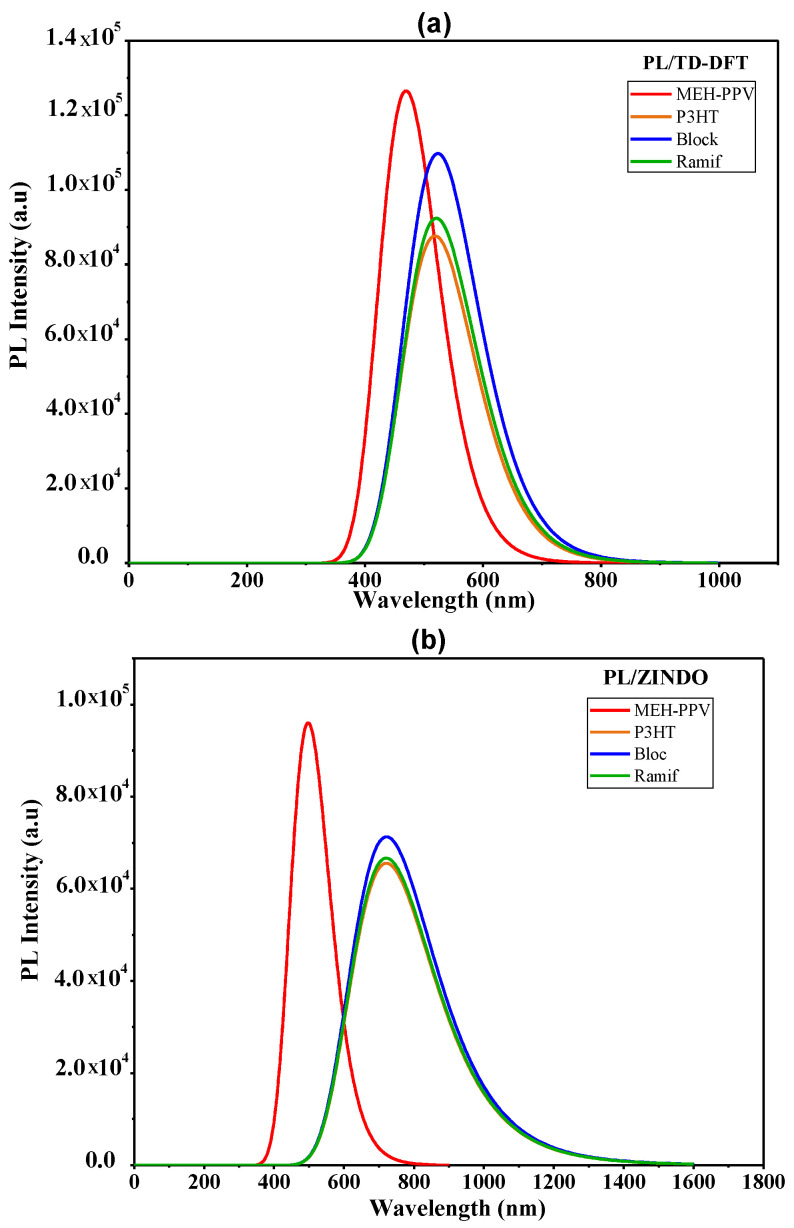
Photoluminescence spectra of MEH-PPV, P3HT, and Block and Ramif copolymer: (**a**) TD-DFT calculations, (**b**) ZINDO calculations.

In Figure 10, we represent the photoluminescence spectra of the two copolymers and those of the basic polymers calculated by the two methods TD-DFT and ZINDO. The values of the maximum emission are summarized in Table 8.

According to this table and the photoluminescence spectra of these copolymers and the basic homopolymers, it is noted that the maximum emissions of the graft copolymer (Ramif) given by the TD-DFT and ZINDO methods are very near to that of P3HT. However, the photoluminescence spectra of the two copolymers present a redshift compared to the MEH-PPV.

These variations explain the great contribution of P3HT and confirm the hypothesis of rupture of MEH-PPV conjugation.

We summarize in Table 9 the characteristics of each transition of these polymers, such as emission energy, oscillator strength, the electronic assignments, and the radiative life time τ, which is estimated by the Einstein transition probabilities [46]:

(2) $$\tau =\frac{c^{3}}{2{\left(E_{flu}\right)}^{2}f}$$

The results obtained from the MEH-PPV-P3HT copolymer for each architecture exhibit interesting properties, such as the visible absorbance range, the low gap, and the charge transfer, which leads to use this as an active layer in the organic photovoltaic cell. 

The active layer is formed by a composite, which is the mixing of a donor material with an acceptor material. In this study, we considered the copolymer MEH-PPV-P3HT as a donor material, while we choose the PCBM as an acceptor material, which is widely used in this device [47].

### 3.6. MEH-PPV-P3HT: PCBM Active Layer Modeling

The modeling of the composite was performed using the previous model structures of the two copolymers MEH-PPV-P3HT. As a start, we optimized the structure of the PCBM to add it with the optimized model structures of the graft copolymer and block copolymer MEH-PPV-P3HT. These calculations were simulated using the DFT/B3LYP/6-31G (d, p). 

**Figure 11:**
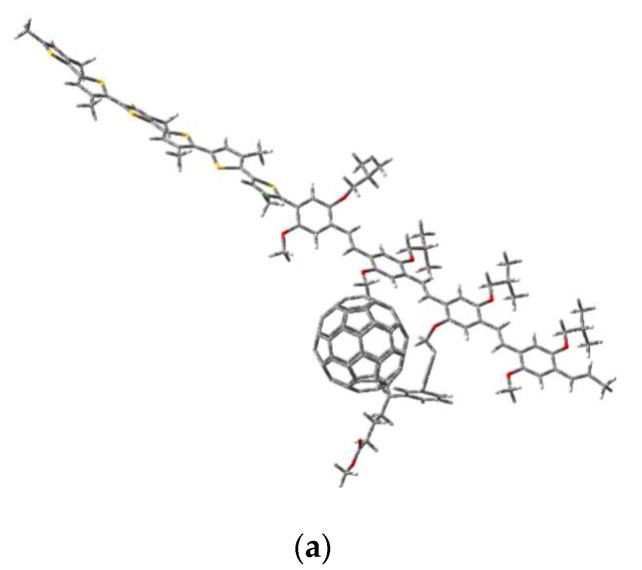

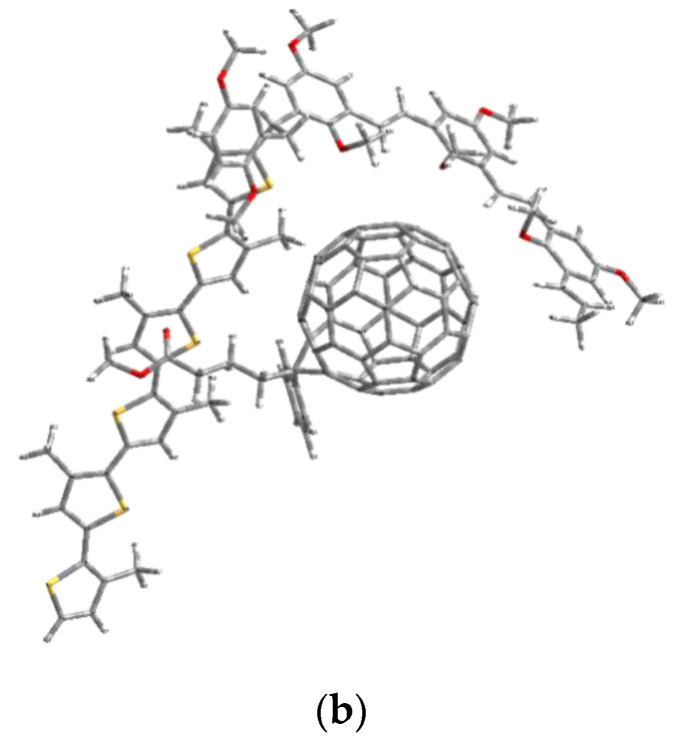
Geometric structures of composites (**a**) MEH-PPV-P3HT (Block): PCBM and (**b**) MEH-PPV-P3HT (Ramif): PCBM.

Figure 11 shows the model structures of our composites MEH-PPV-P3HT: PCBM.

Table 10 summarizes the electronic properties of these composites. 

These results show that the two composites have similar electronic properties, where the composite based on a block copolymer has energies equal to 4.43 eV and 3.06 eV, respectively, of the HOMO and LUMO levels as well as a low gap in the order of 1.37 eV. The composite formed by the graft copolymer (Ramif) has HOMO and LUMO energy levels located, respectively, at 4.42 eV and 3.01 eV, with gap energy equal to 1.41 eV. We notice that there is a reduction in the electronic gap, going from 2.46 eV to 1.37 eV, in the case of a block copolymer. Thus, in the case of a graft copolymer, the gap is reduced by 2.72 eV to 1.41 eV. This reduction is the reflection of the addition of PCBM, which serves to facilitate the charge transport.

**Figure 12:**
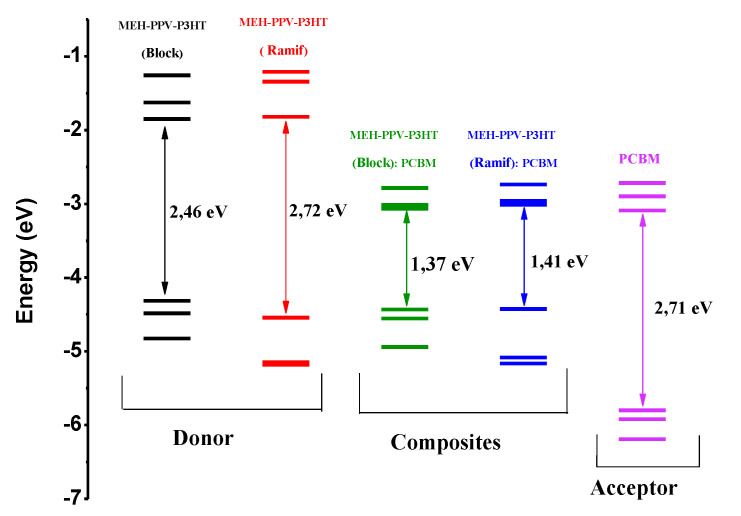
Electronic structures of donor, acceptor, and composites.

The electronic analysis of these compounds elucidates that MEH-PPV-P3HT copolymers are electron donors and hole conductors as well as PCBM is electron acceptor and hole conductor. For further details, we present in Figure 12 the electronic structures of these compounds.

**Figure 13:**
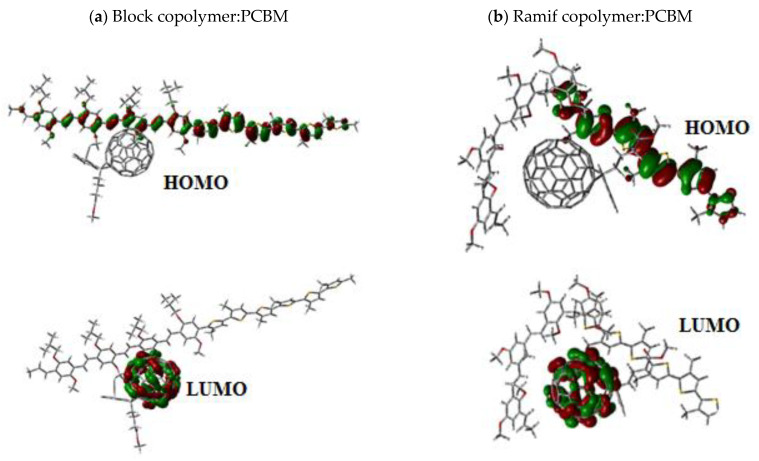
Molecular orbitals of HOMO and LUMO levels of the composites (**a**) block copolymer:PCBM and (**b**) Ramif copolymer:PCBM.

We represent in Figure 13 the molecular orbitals of HOMO and LUMO levels in the ground state of the two composites.

The analysis of the molecular orbital shows that the electron density is localized on the copolymer MEH-PPV-P3HT for the HOMO level. However, the electron density in the LUMO level is situated in the PCBM.

**Figure 14:**
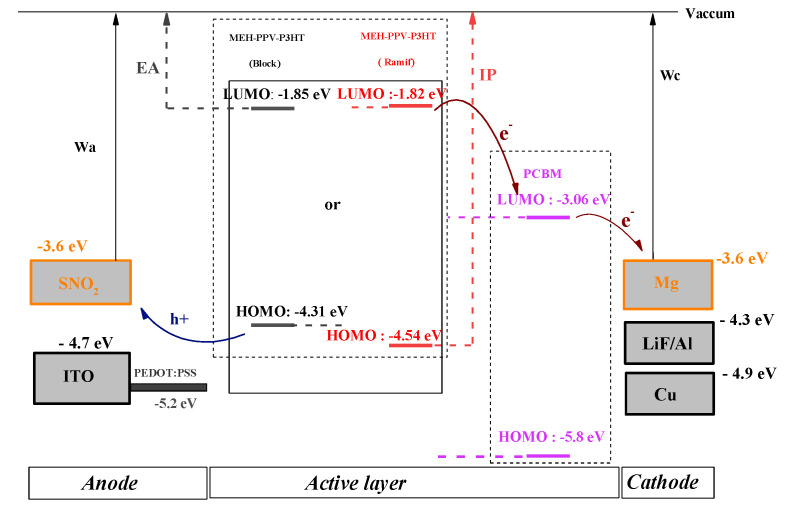
Energy diagram of bilayer cell ITO, SnO_2_/PEDOT: PSS/MEH-PPV-P3HT: PCBM/LiF/Al, Cu, Mg.

**Figure 15:**
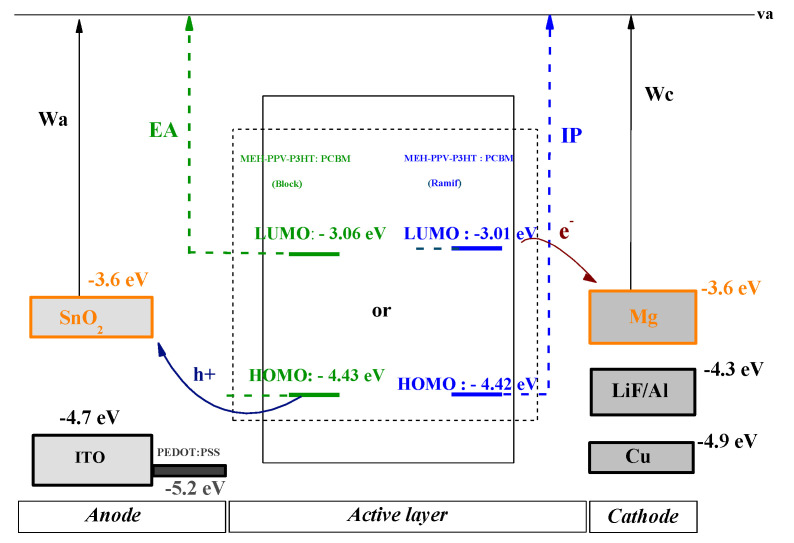
Energy diagram of monolayer cell ITO, SnO_2_/PEDOT: PSS/MEH-PPV-P3HT: PCBM/LiF/Al, Cu, Mg.

In order to present the energy diagram of the composite-based organic photovoltaic cell (MEH-PPV-P3HT: PCBM), different anodes and cathodes (ITO, SnO_2_, LiF/Al, Cu, Mg) were tested to choose those with the best performance. Since there are two types of organic photovoltaic cells, monolayer and bilayer, the energy diagrams of these cells are illustrated, respectively, in the following figures (Figure 14 and Figure 15).

Electronic Affinity (EA) and Ionization Potential (IP) are the electron affinity and ionization potential of donor materials, respectively. The values of these variables are given by the electronic parameters and E_LUMO_ based on Koopman’s theorem: EA = −E_LUMO_ and IP = −E_HOMO_ [48].

From these two diagrams, it can be shown that Mg and SnO_2_ are the most suitable materials to be used as cathode and anode, respectively.

## 4. Conclusions

To summarize, the copolymers based on MEH-PPV and P3HT and the structures, conformation, and vibrational properties were studied using the DFT method. The optical absorption and photoluminescence are simulated in the excited state by the TD-DFT and ZINDO. From these results, we deduced that the coupling of MEH-PPV and P3HT under a block architecture improves the electronic and optical properties of the original compounds through interaction and a charge transfer due to the extension of conjugation. An enlargement of the absorption spectrum and a reduction of the electronic and optical gap are the consequences of the MEH-PPV/P3HT combination. On the other hand, the assembly of MEH-PPV and P3HT under the graft architecture implies that the electronic properties are close to that of P3HT; this can be explained by the rupture of MEH-PPV conjugation. Thus, this copolymer exhibits properties that allow it to be used as an active layer in organic photovoltaic cells.

## Figures and Tables

**Table 1:** Electronic properties of P3HT.

	HOMO (eV)	LUMO (eV)	$E_{g}^{éle}\;(\mathrm{eV})$	λ (nm)	
				TD-DFT	ZINDO
1MEH-PPV	4.99	0.39	4.6	278	317
2MEH-PPV	4.59	1.10	3.49	352	387
3MEH-PPV	4.39	1.38	3.01	398	424
4MEH-PPV	4.29	1.52	2.77	425	443

**Table 2:** Electronic properties of MEH-PPV.

	HOMO (eV)	LUMO (eV)	$E_{g}^{éle}\;(\mathrm{eV})$	λ (nm)	
				TD-DFT	ZINDO
1P3HT	5.64	0.069	5.57	225	299
2P3HT	5.12	0.85	4.27	300	395
3P3HT	4.71	1.39	3.32	377	500
4P3HT	4.58	1.63	2.95	421	557
5P3HT	4.57	1.83	2.74	448	595
6P3HT	4.50	1.93	2.57	470	625

**Table 3:** Vibration IR modes for the graft copolymer (Ramif) and block copolymer.

**IR Ramif**	
**Wavenumber (cm^−1^)**	**Vibration Mode**
846	C=C and C–H wagging in P3HT
1060	CH–CH2 twisting in P3HT
1094	C–H scissoring in PPV
1267	C–H rocking of P3HT and MEH-PPV
1333	C–H twisting of vinyl group + scissoring of phenyl group
1514	C–H rocking and twisting of phenyl group
1562	C=C asymmetric stretching in P3HT
1645	Asymmetric stretching of phenyl group
**IR Block**	
**Wavenumber (cm^−1)^**	**Vibration mode**
1016	C–H twisting in vinyl group
1080	Out-of-plane rocking of phenyl group
1248	C–H symmetrical stretching in-plane of PPV
1273	C–H rocking in vinyl group
1377	Asymmetric stretching of phenyl + C–H rocking in vinyl
1427	CH3 symmetric stretching in P3HT
1458	Phenyl and C–H symmetric stretching in MEH-PPV
1551	C= scissoring in phenyl + C–H rocking in phenyl group
1559	C=C symmetric stretching in phenyl and thiophene

**Table 4:** Vibration Raman modes for the graft copolymer (Ramif) and block copolymer.

**Raman Ramif**	
**Wavenumber (cm^−1^)**	**Vibration Mode**
1164	C–H asymmetric stretching in PPV
1308	Scissoring in-plane of phenyl in MEH-PPV
1401	P3HT deformation
1479	Thiophene scissoring
1570	Twisting of PPV and thiophene
1633	Out-of-plane rocking of PPV
**Raman Block**	
**Wavenumber (cm^−1^)**	**Vibration mode**
1007	CH–CH2 asymmetric stretching in P3HT
1217	C–H rocking in P3HT and MEH-PPV
1402	C=C scissoring in polythiophene PT
1483	Symmetric stretching of thiophene
1493	C=C asymmetric stretching in thiophene
1687	C=C scissoring in vinylene group in MEH-PPV

**Table 5:** Electronic properties of the Block and Ramif copolymers.

Polymer	HOMO (eV)	LUMO (eV)	$E_{g}^{éle}\;(\mathrm{eV})$
4MEH-PPV	4.29	1.52	2.77
6P3HT	4.50	1.93	2.57
4MEH-PPV-6P3HT (Ramif)	4.54	1.82	2.72
4MEH-PPV-6P3HT (Block)	4.31	1.85	2.46

**Table 6:** Electronic transitions of the optical absorption of the basic polymers and their derived copolymers.

	Polymer	Transition	λ (nm)	E (eV)	f	Assignment; H = HOMO, L = LUMO
TD-DFT	4MEH-PPV	$S_{0}\to S_{1}$	425.7	2.91	3.2642	H0->L+0(+85%) H1->L+1(+9%)
	6P3HT	$S_{0}\to S_{1}$	470.7	2.63	2.2636	H0->L+0(+88%) H1->L+1(7%)
	Ramif	$S_{0}\to S_{1}$	445.9	2.78	2.3700	H0->L+0(+86%) H1->L+2(7%)
	Block	$S_{0}\to S_{1}$	469.5	2.64	4.4501	H0->L+0(+44%)H1-> L+0(+29%) H1->L+1(11%)
ZINDO	4MEH-PPV	$S_{0}\to S_{1}$	443.1	2.80	2.6780	H0->L+0(+75%) H1-> L+1(+14%)
	6P3HT	$S_{0}\to S_{1}$	625.6	1.98	1.7946	H0->L+0(+81%) H1-> L+1(11%)
	Ramif	$S_{0}\to S_{1}$	585.9	2.12	1.7458	H0->L+0(+77%) H1-> L+1(10%)
	Block	$S_{0}\to S_{1}$	610	2.03	2.0843	H0->L+0(+75%) H2-> L+2(+5%)

**Table 7:** The optical gap energy of MEH-PPV, P3HT, and Block and Ramif copolymer.

Polymer	$E_{g}^{opt}\;(\mathrm{eV})\;(\mathrm{TD}-\mathrm{DFT})$	$E_{g}^{opt}\;(\mathrm{eV})\;(\mathrm{ZINDO})$	$E_{g\;exp}^{opt}\;(\mathrm{eV})$
MEH-PPV	2.33	2.19	2.2 [44]
P3HT	2.03	1.4	1.9–2.1 [45]
Block	2	1.49	–
Ramif	2.64	2.17	–

**Table 8:** The maximum emission of MEH-PPV, P3HT, and Block and Ramif copolymer.

Polymer	λ(nm) TD−DFT	λ(nm) ZINDO
MEH-PPV	469.5	497.6
P3HT	518.5	721.3
MEH-PPV-P3HT (Block)	523.4	721.8
MEH-PPV-P3HT (Ramif)	520.7	721.1

**Table 9:** Electronic transitions of the emission of the basic polymers and their derived copolymers.

	Polymer	Transition	λ(nm)	E (eV)	f	τ (ns)	Assignment; H = HOMO, L = LUMO
TD-DFT	MEH-PPV	$S_{0}\to S_{1}$	469.5	2.64	3.12	1.39	H0->L+0(+91%) H-1-> L+1(+5%)
	P3HT	$S_{0}\to S_{1}$	518.6	2.39	2.16	2.23	H0->L+0(+93%)
	Ramif	$S_{0}\to S_{1}$	520.7	2.38	2.2819	2.12	H0->L+0(+92%)
	Block	$S_{0}\to S_{1}$	523.4	2.37	2.7115	1.79	H0->L+0(+90%)
ZINDO	MEH-PPV	$S_{0}\to S_{1}$	497.6	2.49	2.37	1.95	H0->L+0(+84%) H-1-> L+1(+7%)
	P3HT	$S_{0}\to S_{1}$	721.3	1.72	1.61	4.16	H0->L+0(+87%) H-1-> L+1(6%)
	Ramif	$S_{0}\to S_{1}$	721.1	1.72	1.6460	4.08	H0->L+0(+86%) H-1-> L+1(+5%)
	Block	$S_{0}\to S_{1}$	721.8	1.72	1.7651	3.80	H0->L+0(+86%)

**Table 10:** Electronic properties of composites.

Composite	HOMO (eV)	LUMO (eV)	$E_{g}^{éle}\;(\mathrm{eV})$
MEH-PPV-P3HT (Block): PCBM	4.43	3.06	1.37
MEH-PPV-P3HT (Ramif): PCBM	4.42	3.01	1.41
MEH-PPV-P3HT (Block)	4.31	1.85	2.46
MEH-PPV-P3HT (Ramif)	4.54	1.82	2.72
PCBM	5.8	3.09	2.71

## Data Availability

Exclude this statement if the study did not report any data.
